# Something Practical about Micrococci

**Published:** 1883-09

**Authors:** 


### Something Practical About Micrococci.

"I care not," said a gentleman the other day, who is well known for the practical nature of his contributions to medical science; "I care not whether micrococci are the cause *per se*, or whether they are the vehicles carrying the cause, of zymotic diseases, but I do know one thing—they possess great prognostic value."

He then went on to describe his belief in the following proposition: That in scarlet fever, measles, puerperal fever, and the like, when a microscopic examination of the blood demonstrated the presence of large quantities of granular matter (call them micrococci, or whatever you choose), the prognosis is very bad.

This matter, he believes, acts mechanically, it obstructs the capillary circulation, causing the formation of heart-clot, and death from mechanical interference with the circulation.

In support of this view, he cites numerous instances where this granular matter has been found in the blood, when, after death, enormous clots are found in the right ventricle, while the left ventricle is found firmly contracted, dying in systole, failing to overcome the mechanical obstruction offered by this granular matter in the capillaries.

When he finds this condition under the microscope, his prognosis is grave, and *vice versa*.

The discoloration of the skin, found after death in these so-called malignant cases, he considers due to this capillary stasis.

Still further, and what is of greater moment, he is convinced that alcohol possesses great power to alter this morbid condition of the blood. "Make your patients drunk, if you can, but you will find that the tolerance of alcohol in these cases is something wonderful. I used to use carbonate of soda and digitalis, and lost my patients; I now use whiskey, and save them." As soon as he has reason to suspect a grave case, from the severity of the symptoms, he commences to use alcohol and pushes it to the stage of intoxication, and he has had most excellent results.

Our authority is so well worthy of confidence that we would be glad to have his recommendations heeded and to have our readers give the alcohol treatment of grave zymotic diseases a fair trial.

It is truly refreshing in these days of ponderous, wordy, theoretical, micrococcal discussions, to have some reliable authority tell us something truly practical about them.—*Medical and Surgical Reporter.*

